# Exploring polypharmacy burden among elderly patients with chronic diseases in Chinese community: a cross-sectional study

**DOI:** 10.1186/s12877-021-02247-1

**Published:** 2021-05-13

**Authors:** Yongli Wang, Xiaodan Li, Dongmei Jia, Beilei Lin, Bo Fu, Bei Qi, Zhenxiang Zhang

**Affiliations:** 1 School of Nursing and Health, Zhengzhou University, No.100 Science Avenue, Henan Zhengzhou, China; 2 Peking University People’s Hospital, Xicheng Dist, Beijing, China

**Keywords:** Medicine, Medicine burden, Patient‐reported outcome, Multi‐morbidity, Living with Medicines Questionnaire

## Abstract

**Background:**

In the long-term use of multiple medications for elderly patients diagnosed with chronic diseases, medication problems are prominent, which seriously reduces their quality of life. The burden of medications of patients critically affects their medication beliefs, behaviors and disease outcomes. It may be a solution to stress the burden of medications of patients. Its medication issues develops a novel perspective. The present study aimed to exploit the Chinese version of Living with Medicines Questionnaire-3(C-LMQ-3) to quantify the medicines burden of elderly patients diagnosed with chronic diseases in China, and evaluate the relevant demographic characteristics of sub-populations with high medicines burden.

**Methods:**

The survey was distributed to elderly patients aged ≥ 60 years with chronic disease by using ≥ 5 medicines, C-LMQ-3 scores and domain scores were compared by the characteristics of elderly patients by employing descriptive statistics and performing statistical tests.

**Results:**

On the whole, 430 responses were analyzed, and the participants were aged between 60 and 91 years, with the average age of 73.57 years (SD: 7.87). Most of the responses were female (61.7 %) with middle school education (38.5 %). Moreover, 54.1 % of the participants lived with spouse only, 16.2 % had both spouse and children, and 10.0 % lived alone. As indicated from regression analysis, higher C-LMQ-3 scores were associated with those who were with low education level, 60–69 years-old, using ≥ 11 medicines, using medicines ≥ 3 times a day, income per month (RMB) ≤ 3000, and who having higher monthly self-paid medication (RMB) ≥ 300 (*p* < 0.01). Burden was mainly driven by cost-related burden, concerns about medicines, and the lack of autonomy over medicine regimens.

**Conclusions:**

This study presents the preliminary evidence to elderly patients diagnosed with chronic diseases in mainland China that pay attention to multiple medications burden may help reduce the Drug Related Problems, whereas some elderly patients have a higher burden of medication. Chinese health care providers are required to primarily evaluate and highlight such patients, and formulate relevant intervention strategies to ensure medication adherence and daily medication management of elderly patients with polypharmacy.

## Introduction

In 2009, the World Health Organization (WHO) defined “multi-morbidity” as having two or more chronic diseases. Multi-morbidity is recognized as a common problem, mainly occurring in the elderly. One of the reasons is that population ageing is a global phenomenon [1]. As reported from the existing study, 61.7-86.3 % of the elderly suffer from different chronic diseases in China [2–4]. However, according to the recently achieved data from the China Health and Retirement Longitudinal Study, physical multi-morbidity increased with age and was more common in poorer regions [5]. As suggested from the relevant literature on the multi-morbidity of chronic diseases among the elderly in China [2, 6, 7], the most common six kinds of chronic diseases were found, including hypertension, diabetes mellitus, coronary heart disease, stroke, hyperlipidemia and chronic obstructive pulmonary disease (COPD).

Medicines are the most commonly used medical technology to treat chronic diseases, and elderly patients often need to use multiple medicines due to multiple chronic diseases [8–10]. As reported from literature, ≥ 5 medicines are the most commonly used definition of polypharmacy [8, 11]. Polypharmacy contributes to increases the risk for a Drug Related Problems (DRPs) (e.g., adverse medication events [12], decreased medication adherence [13], increased hospitalization [14],cognitive impairment, falls and increased mortality [15]). As revealed from a systematic review [16], medication burden acts as a core factor affecting a patient’s beliefs about medication adherence and health status, and to some extent reflects the patient’s attitude and willingness and ability to handle medication use [14, 17]. Therefore, measurement of medication burden may provide an important perspective for helping to reduce these medication-related problems and improve adherence.

Recently, as polypharmacy becomes widespread, a tool has been developed and validated by Krska et al. in the UK, which specifically aims to measure medicine-related burden in daily life-the Living with Medicines Questionnaire (LMQ) [18–20].The LMQ originated from qualitative interviews with patients who were diagnosed with multi-morbidity and using multiple medicines [21]. The LMQ can assess the patient’s medicine-related burden (e.g., side effects, effectiveness, general concerns, and cost burden [20]). As indicated from existing studies, the tool exhibits high reliability and effectiveness, and has been employed in numerous nations (e.g., Australia, Belgium, the Netherlands, New Zealand, Qatar and the United Kingdom) [22–25]. Furthermore, the instrument has also been translated and validated for use in an elderly Chinese population- the C-LMQ-3 [26].

There are very few studies in China that report experiences of medication burden for elderly patients with multi-morbidity using multiple medicines. Research studies on both multi-morbidity and the burden of medication for patients with chronic diseases shift their stress to economic aspects of treatment burden [27]. Medicine burden refers to a different construct, and rare studies in China have explored the burden which medicines can impose on peoples’ daily lives (e.g., side effects and other interferences [27]). For this reason, this study aimed to use C-LMQ-3 to quantify the medicines burden of elderly patients diagnosed with chronic diseases in China, as well as assess the relevant socio demographic characteristics of subpopulations with high medicines burden.

## Materials and methods

### Setting, Study Population, and Sample selection

The present study was a cross-sectional correlational design. Potential participants were recruited based on convenience sampling in Zhengzhou from January to August 2020. Eligible participants were identified by screening residents’ health records in three community health service institutions to ensure that they had at least two chronic diseases. The following inclusion criteria were employed: (1) aged ≥ 60 years (abiding by the Law of the People’s Republic of China on the Protection of the Rights and Interests of the Elderly); (2) diagnosed with at least two chronic diseases (chronic diseases including hypertension, diabetes mellitus, coronary heart disease, stroke, hyperlipidemia and COPD); (3) using ≥ 5 medications for more than 3 months; and (4) willing to participate in this study and provide informed consent. Individuals with cognitive impairments or other serious physical diseases who were unable to respond appropriately to the investigation were excluded from the study.

To ensure adequate statistical power, the sample size was calculated with the G*Power 3.1 in a multiple regression analysis with power set at 0.80, α = 0.05 [28]. A medium effect size was 0.15 [29], and the number of dependent variables was 12. The resultant sample size was at least 148 samples required, leading to a 20 % inefficiency rate.

### The survey Tool

This study used the C-LMQ-3 translated by the team of this study [26], which consisted of 39 items in eight domains (i.e., relationships with health professionals, practicalities, interferences, effectiveness, side effects, concerns, cost and autonomy, scored at a 5-point scale from strongly agree to strongly disagree). Total LMQ-3 scores range from 39 to 195; higher scores indicate greater burden associated with medicine use. The Cronbach’s alpha for C-LMQ-3 was reported as 0.855 in elderly patients diagnosed with chronic diseases. The C-LMQ-3 also includes a visual analogue scale (VAS), which measures global burden scored from 0 to 10.

### Data Collection

Data were collected during community elderly health checkups, free consultations, health lectures or home visits. The investigators were trained in the purpose of the investigation, the method of using the questionnaire, and the precautions. Data were collected by eight investigators, four researchers, and four community-medical staff. The questionnaire was filled out independently by the survey participants. The questionnaire issuer was permitted to read out the contents of the questionnaire separately to help respondents complete the questionnaire, and check whether there were any problems (e.g., omissions and missing responses). Furthermore, all the questionnaires were returned on the spot after completion.

### Statistical analysis

All data were inputted into Microsoft Office Excel 2016 software, and IBM SPSS 21.0 was used to conduct the statistical analysis. Mean, Standard Deviation (SD), frequencies were adopted to describe the demographic characteristics of elderly patients with chronic disease. Based on analysis of variance and t-test, the multiple medicine related-burden level of the participants was analyzed. Multiple linear regression analysis was used to analyze the factors of multiple medicines related- burden.

## Results

### General Characteristics of patients

Among the 527 eligible patients identified, a total of 469 patients in the initial study population agreed to be surveyed, and 453 (96.6 %) responded to the survey. A completion rate of 94.9 % (*n* = 430) was gained after excluding those who provided an incomplete survey (*n* = 23, 5.1 %) and those who withdrew from the halfway survey (*n* = 16, 3.4 %). The common reasons for withdrew from the halfway survey involved a lack of time (*n* = 3), a lack of interest (*n* = 9) and fear of disclosure of personal health information (*n* = 4).

The demographic and medical characteristics of the 430 respondents are listed in Table 1. The participants were aged between 60 and 91 years, and the average age was 73.57 years (SD: 7.87). Most were female (61.7 %) and had a middle school education (38.5 %). Furthermore, 54.0 % of the participants had a monthly income less than 3000 Renminbi (RMB). Moreover, 54.1 % of the participants lived with spouse only, 16.2 % with both spouse and children, and 10.0 % lived alone. On average, participants took 10.1 ± 3.5 prescription medications, ranging from 5 (*n* = 52) to 18 (*n* = 1), the median value of the prescribed medication by patients was 11. Most used 5–10 medicines (n = 174, 40.4 %) or 11–15 medicines (203, 47.1 %) and used medicines twice (91, 21.1 %) or three times daily (224, 52.0 %).

**Table 1:** Demographic characteristics of participants (*n* = 430)

Characteristic	n(%)				score(*`x ± s*)		*t/F* value	*P*
Age(years)								
60~	116(27.0)				113.53 ± 9.39		7.624^b^	0.001
70~	216(50.2)				114.34 ± 11.80			
80~	98(22.8)				109.13 ± 11.39			
Gender								
Male	164(38.1)				112.17 ± 11.08		-1.094^a^	0.274
Female	266(61.7)				113.39 ± 11.38			
Education level								
Primary school or less	69(16.0)				116.62 ± 10.29		12.168 ^b^	<0.001
Middle school	166(38.5)				115.28 ± 10.35			
High school	136(31.6)				110.45 ± 11.02			
College or above	59(13.7)				107.71 ± 12.41			
Marital status								
Unmarried	3(0.7)				118.67 ± 7.51		1.136^b^	0.334
Married	313(72.6)				112.99 ± 11.01			
Divorce	10(2.3)				107.20 ± 11.60			
Widowed	104(24.1)				113.13 ± 12.02			
Primary caregiver								
Spouse	138(32.0)				112.12 ± 8.84		2.984 ^b^	0.019
Child	154(35.7)				111.53 ± 10.21			
Nursing workers	39(9.0)				114.67 ± 15.15			
No	86(20.0)				116.22 ± 13.63			
Other	13(3.0)				111.00 ± 12.28			
Income per month(RMB)								
<2000	83(19.3)				116.93 ± 10.13		17.315 ^b^	<0.001
2000~	149(34.6)				115.83 ± 11.20			
3000~	135(31.3)				110.01 ± 10.06			
>4000	63(14.6)				107.03 ± 11.37			
Living status								
Lives with spouse only	233(54.1)				113.31 ± 10.23		0.199 ^b^	0.897
Lives with children only	84(19.5)				112.58 ± 12.46			
Lives with both spouse and children	70(16.2)				112.33 ± 11.86			
Lives alone	43(10.0)				112.51 ± 13.38			
Health conditions(diagnoses)								
High blood pressure	362(84.0)				112.95 ± 11.37		-0.095	0.924
Stroke	199(46.2)				113.20 ± 11.22		-0.466	0.641
Diabetes	255(59.2)				113.73 ± 11.22		-1.794	0.074
Hyperlipidemia	281(65.2)				111.99 ± 19.70		2.385	0.022
Coronary heart disease	254(58.9)				112.30 ± 10.74		1.383	0.176
Chronic obstructive pulmonary disease	134(31.1)				115.22 ± 10.72		-2.867	0.004
Number of medicines								
5 ~ 10	174(40.4))				109.71 ± 10.31		25.901 ^b^	<0.001
11 ~ 15	203(47.1))				113.41 ± 11.15			
>15	53(12.3)				121.66 ± 9.86			
Frequency of medicines								
Once per day	49(11.4)				105.16 ± 9.97		16.367 ^b^	<0.001
Twice per day	91(21.1)				110.36 ± 11.49			
Three per day	224(52.0)				114.16 ± 10.71			
More than three times per day		66(15.3)			118.06 ± 10.09			
Monthly self-paid medication(RMB)								
<100	44(10.2)				104.20 ± 9.69		13.573^b^	<0.001
100~	98(22.7)				110.28 ± 9.93			
300~	128(29.7)				113.64 ± 11.57			
500~	129(29.9)				115.89 ± 10.12			
800~	31(7.2)				118.42 ± 12.37			

Tips: ^a^ represents T value; ^b^ represents *F* value

### Assessment of Polypharmacy Burden

Table 2 lists the responses to individual statements in the C-LMQ-3 and 8 domains score, the C-LMQ-3 total scores were normally distributed, i.e., mean 112.9 (SD = 11.3), range 84–145 (maximum possible range 39–195). VAS scores were skewed to higher values with a median (range) of 6 (0–10) and mean 5.4 (SD = 1.7). Moreover, the VAS‐burden scores displayed a strong-positive relationship with C-LMQ‐3 total scores (Spearman’s r = 0.869; *p* < 0.001). The top 10 items with the maximal scores in C-LMQ-3 are listed in Table 3.

**Table 2:** Responses to individual statements in the C-LMQ-3 and 8 domains score (*n* = 430)

Statements /domains	Agree/ Strongly Agree N (%)	Neutral opinion N (%)	Disagree/ Strongly Disagree N (%)
**Practical difficulties (**6 items; Mean (SD) = 17.2 (2.5)**)**			
I find getting my prescriptions from the doctor difficult	113 (26.2)	134(31.2)	183(42.6)
I am comfortable with the times I should take my medicines	262(60.9)	93(21.6)	75(17.5)
I am concerned that I may forget to take my medicines	207(48.1)	132(30.7)	91(21.2)
It is easy to keep my medicines routine	250(58.1)	123(28.6)	57(13.3)
I find using my medicines difficult (e.g.: taking the medication from the package, keeping in mind the precautions for medication use, etc.).	216(50.2)	71(16.5)	143(33.3)
I have to put a lot of planning and thought into taking my medicines	107(24.9)	163(37.9)	160(37.2)
**lack of effectiveness (5 items**; Mean (SD) = 13.3 (2.6)**)**			
My medicines prevent my condition getting worse	219(50.9)	160(37.2)	51(11.9)
My medicines live up to my expectations	208(48.4)	164(38.1)	58(13.5)
My medicines allow me to live my life as I want to	205(47.7)	102(23.7)	123(18.6)
My medicines are working	232(54.0)	114(26.5)	84(19.5)
The side effects are worth it for the benefits I get from my medicines	181(42.1)	159(37.0)	90(20.9)
**Cost-related burden (3 items**; Mean (SD) = 9.4 (2.3)**)**			
I worry about paying for my medicines	229(53.3)	91(21.1)	110(25.6)
I sometimes have to choose between buying basic essentials or medicines	103(24.0)	143(33.3)	184(42.8)
I have to pay more than I can afford for my medicines.	167(38.8)	161(37.4)	102(23.8)
**Communication/relationships with HCPs (5 items**; Mean (SD) = 13.6 (3.4)**)**			
I trust the judgement of my doctor(s) in choosing medicines for me.	293(68.1)	49(11.4)	88(20.5)
My doctor(s) listen to my opinions about my medicines	218(50.7)	99(23.0)	113(26.3)
My doctor takes my concerns about side effects seriously.	164(38.1)	145(33.7)	121(28.1)
I get enough information about my medicines from my doctor(s)	256(59.5)	90(21.0)	84(19.5)
The health professionals providing my care know enough about me and my medicines	159(37.0)	160(37.2)	111(25.8)
**Concerns about medicine use (7 items**; Mean (SD) = 21.8 (4.1)**)**			
I worry that I have to take several medicines at the same time	213(49.5)	73(17.0)	144(33.5)
I would like more say in the brands of medicines I use	203(47.2)	70(16.3)	157(36.5)
I feel I need more information about my medicines	204(47.4)	138(32.1)	88(20.5)
I am concerned about possible damaging long-term effects of taking medicines	194(45.1)	50(11.6)	186(43.3)
I am concerned that I am too reliant on my medicines	178(41.4)	129(30.0)	123(28.6)
I am concerned that my medicines interact with eating habits (other food, alcohol, drinks, etc.).	101(23.5)	129(30.0)	200(46.5)
I worry that my medicines may interact with each other	150(34.9)	171(39.8)	109(25.3)
**Side-effect-burden (4 items**; Mean (SD) = 11.9 (1.9)**)**			
The side effects I get are sometimes worse than the problems for which I take my medicines	205(47.7)	152(35.3)	73(17.0)
The side effects that I get from my medicines interfere with my day to day life	143(33.3)	149(34.7)	138(32.1)
The side effects I get from my medicines are bothersome	147(34.2)	173(40.2)	110(25.6)
The side effects I get from my medicines adversely affect my wellbeing	70(16.3)	106(24.6)	254(59.1)
**Interference to day-to-day life (6 items**; Mean (SD) = 17.4 (2.6)**)**			
My medicines interfere with my social or leisure activities	109(25.3)	256(59.6)	65(15.1)
Taking medicines affects my going out (walking, Cycling, driving, etc.)	81(18.8)	159(37.0)	190(44.2)
My medicines interfere with my social relationships with (family, friends, colleagues)	64(14.9)	132(30.7)	234(54.4)
Taking medicines causes problems with daily tasks	135(31.4)	218(50.7)	77(17.9)
My medicines interfere with my sexual life	79(18.4)	135(31.4)	216(50.2)
My life revolves around using medicines	170(39.5)	193(44.9)	67(15.6)
**Autonomy/control (3 items**; Mean (SD) = 8.5 (2.2)**)**			
I can vary the dose of the medicines I take	231(53.7)	133(30.9)	66(15.4)
I can choose whether or not to take my medicines	181(42.1)	100(23.3)	149(43.6)
I can vary the times I take my medicines	154(35.8)	147(34.2)	129(30.0)

**Table 3:** The top 10 items with the highest scores in C-LMQ-3

Item	score(`*x* ± *s*)	sort
3. I worry about paying for my medicines	3.37 ± 1.05	1
7. I feel I need more information about my medicines	3.37 ± 0.94	2
19. The side effects I get are sometimes worse than the problems for which I take my medicines	3.33 ± 0.78	3
8. I am concerned that I may forget to take my medicines	3.31 ± 0.92	4
39. My life revolves around using medicines	3.28 ± 0.78	5
4. I worry that I have to take several medicines at the same time	3.24 ± 1.07	6
31. I have to pay more than I can afford for my medicines.	3.22 ± 0.94	7
27. I find using my medicines difficult (e.g.: taking the medication from the package, keeping in mind the precautions for medication use, etc.).	3.20 ± 0.95	8
6. I would like more say in the brands of medicines I use	3.18 ± 1.24	9
34. My medicines interfere with my social or leisure activities	3.16 ± 0.76	10

### Factors associated with polypharmacy burden

Table 1 presents the results of the differences in C-LMQ-3 scores between the demographic characteristics and medicine use of different elderly patients diagnosed with chronic diseases. Significant differences were identified in the age, education level, primary caregiver, income per month (RMB), types of medicine, frequency of medicines, monthly self-paid medication (RMB) and burden of multiple medications among elderly patients diagnosed with chronic diseases (*P* < 0.05). No significant differences were reported in burden of multiple medications among elderly patients diagnosed with chronic diseases regarding gender, marital status, living status, and health conditions (*P* > 0.05).

Table 4 lists the independent variable assignment in the fitted multiple linear regression model. Table 5 shows results from the multiple linear stepwise regression analysis to determine factors associated with multiple medicines burden of elderly patients diagnosed with chronic diseases. Age, education level, primary caregiver, income per month (RMB), number of medicines, frequency of medicine use, monthly self-paid medication (RMB) displayed noticeable relationships to multiple medicines burden. Elderly patients with relatively young age, low education level, low per capita economic income, higher numbers of medicines, high frequency of daily use and high monthly self-payment medication fees may be subject to heavy multiple medicine burden. No multicollinearity was identified here, since the tolerance of each variable was between 0.82 and 0.97, > 0.25, and variance inflation factors for the mentioned variables ranged from 1.03 to 1.22, both < 10.0.

**Table 4:** Description of the assignment of independent variables

Independent variable	Assignment method			
Age(years)	1=60~; 2=70~; 3=80~			
Education	1= Primary school or less; 2= Middle school; 3= High school; 4= College or above			
Income per month(RMB)	1=<2000; 2=2000~; 3=3000~; 4=4000~			
Primary caregiver(Xg)	(1)	(2)	(3)	(4)
Spouse	X1= 1	X2=0	X3=0	X4=0
Child	X1= 0	X2=1	X3=0	X4=0
Nursing workers	X1= 0	X2=0	X3=1	X4=0
No	X1= 0	X2=0	X3=0	X4=1
Other owever, according to the recently achieved data fro	X1= 0	X2=0	X3=0	X4=0
Types of medicines(kinds)	1=5~10; 2=11~15; 3=>15			
Monthly self-paid medication(RMB)	1=<100; 2=100~; 3=300~; 4=500~; 5=800~			
Frequency of medicines	1= Once per day; 2= Twice per day; 3= Three per day; 4= More than three times per day			

Primary caregivers need to set dummy variables

**Table 5:** Multivariable stepwise linear model analysis for the association with the multiple medication burden among elderly patients with chronic diseases (n = 430)^a^

Variables((Reference)	*β*	*95 % CI*	*SE*	*t*	*P* value
Age(per 10 years)	-2.044	(-3.296;-0.793)	0.637	-3.211	0.001
Education(College or above)	-1.960	(-3.010;-0.911)	0.534	-3.671	<0.001
Income per month(RMB>4000)	-1.799	(-2.806;-0.791)	0.513	-3.508	<0.001
Types of medicines(>11)	3.728	(2.381;5.075)	0.685	5.441	<0.001
Monthly self-paid medication(RMB>800)	2.125	(1.293;2.957)	0.423	5.022	<0.001
Frequency of medicines(≥ 3 times daily)	3.213	(2.168;4.258)	0.532	6.043	<0.001
Constant	101.03	(92.956;109.104)	4.108	24.596	0.000

*R*^2^ = 0.345, Adj *R*^2^ = 0.329; *F* = 10.31, *P* < 0.0001

^a^Adjustment for age (60–69,70–79,≥80), education level (Primary school or less, Middle school, High school, College or above), Income per month(RMB) (<2000,2000~,3000~,>4000), Types of medicines (5 ~ 10,11 ~ 15,>15), Frequency of medicines (Once per day, Twice per day, Three per day, More than three times per day), Monthly self-paid medication(RMB)(100,100~,300~,500~,800~)and other variables in the models

## Discussion

In the present study, the C-LMQ-3 was used. It was reported that a relatively heavy burden of multiple medications is imposed on elderly patients diagnosed with chronic diseases in China, the C-LMQ-3 total scores were mean 112.9 ± 11.3, range 84–145 (maximum possible range 39–195). As indicated from domain analysis, the main drivers of burden included (i) cost-related burden (ii) concerns about medicines, as well as (iii) the lack of autonomy over medicine regimens. Multiple linear regression indicated significantly higher burden in patients with low education level, aged 60–69 years, using ≥ 11 medicines, using medicines ≥ 3 times a day, income per month (RMB) ≤ 3000, and having higher monthly self-paid medication (RMB) ≥ 300. In addition, governments, health professionals and pharmacists should place the stress of their interventions on the mentioned populations to reduce their medicines burden and help them make optimal use of their medicines.

As suggested from the results here, participants scored higher on the cost burden domain, which is probably explained by their children’s support. Besides, some medicines are not included in the medical insurance system. This complies with the results of an American survey on the cost of daily medication for 8777 patients ≥ 65 years of age [30]. Thus, despite the long-term multi-drug elderly patients receiving medical insurance, the expensive medicines costs remain related to the medication burden. In addition, China’s basic medical insurance system is impacted by several factors (e.g., economic foundations and institutional settings) and exhibits a certain degree of regionalization. Moreover, some chronic diseases and related medicines are not covered by medical reimbursement. Accordingly, the government should vigorously facilitate the rectification and reform of medical insurance [31], implement innovative outpatient chronic disease management, implement family doctors’ contracted services, and soundly operate chronic disease prescriptions [32].

As revealed from the results of this study, the medication burden was more significant when the participants were aged 60–69 years. This result does not comply with the other studies reporting that those with older ages have lower medication burden [24, 33]. The reason may be that the patients included in this study were aged (73.57 ± 7.87) years, older patients may take medication for a longer period and can more effectively develop a strategy in line with self-medication habits. Furthermore, older patients have relatively low expectations of medicines treatment [14] and have low perception of the impact of medicines interfering with their daily lives. Several patients enjoy medical insurance policy subsidies, and the cost burden of medication is relatively light. Some patients maintain good communication with medical staff [34, 35].

Under higher participants’ educational level and economic income, the patients’ multiple medication burden level was lower, which may be related to their less economic burden, and the patients with higher education level would actively seek other medical resources (e.g., medication help and medication information [36, 37]). Moreover, participants with high education level have higher health literacy and medication literacy level, so the medication adherence can be improved [35, 38].

Complying with the expectation, high or frequent use of medicines was associated with higher C-LMQ-3 scores; multiple linear regression suggested an association (*P*<0.001) and higher scores between the use of ≥ 11 medicines per day or ≥ 3 times a day. The reason may be that when the patients use more types of medicines per day, more time, energy, emotion and money spent on medication practice management [25, 39] (e.g., medicine selection, medicine reserve, medicine-related precautions, medicine collocation taboo, medicine purchase that will aggravate their medication burden [40, 41]).

There are several strengths in this study. First, this is the study to initially investigate the factors of the burden of medication in the long-term multi-medicines use of elderly patients diagnosed with chronic diseases in Chinese mainland. Second, this study assessed the various aspects of medication burden of elderly patients diagnosed with chronic diseases during the long-term use of multiple medicines, which can help gain insights into the daily medication experience of patients. Third, health care providers can draw upon this study to screen chronic diseases patients who require medication and provide interventions to reduce the burden of medication. Fourth, a validated tool of C-LMQ-3 was adopted to measure medication burden, which led to the validity of the study results.

The limitations of this study should be acknowledged. First, a convenience sample of elderly participants with chronic disease was employed which was drawn only from Zhengzhou community in China. Whether and to what extent they can represent other elderly patients with chronic disease population in China requires in-depth investigation. For this reason, the generalizability of our findings may be limited. Second, the sample size here was limited. In subsequent research, multi-center and large-sample studies should be conducted in other regions of China to verify whether the survey results here can be confirmed in other Chinese populations.

## Conclusions

This study presents the preliminary evidence to elderly patients diagnosed with chronic diseases in mainland China, stressing that multiple medications burden may help reduce the drug related problems, especially domains of cost-related burden, concerns about medicines, and the lack of autonomy over medicine regimens. Those elderly patients with low education level, aged 60–69 years, using ≥ 11 medicines, using medicines ≥ 3 times a day, income per month (RMB) ≤ 3000, and monthly self-paid medication (RMB) ≥ 300 have a higher burden of medication. It is therefore suggested that Chinese health care providers should focus on evaluating and stress such patients, and formulate relevant intervention strategies to ensure medication adherence and daily medication management of elderly patients with polypharmacy use.

## Data Availability

The data set used and analyzed in this study can be obtained from the corresponding author according to relevant reasonable requirements.

## Abbreviations

C-LMQ-3: The Chinese version of Living with Medicines Questionnaire-3
WHO: World Health Organization
COPD: Chronic obstructive pulmonary disease
DRPs: Drug Related Problems
LMQ: The Living with Medicines Questionnaire
VAS: Visual analogue scale
RMB: Renminbi
